# Fork- and Comb-like Lipophilic Structures: Different Chemical Approaches to the Synthesis of Oligonucleotides with Multiple Dodecyl Residues

**DOI:** 10.3390/ijms241914637

**Published:** 2023-09-27

**Authors:** Timofey D. Zharkov, Ekaterina M. Mironova, Oleg V. Markov, Sergey A. Zhukov, Svetlana N. Khodyreva, Maxim S. Kupryushkin

**Affiliations:** Institute of Chemical Biology and Fundamental Medicine, Siberian Branch of RAS, Lavrentiev Ave. 8, 630090 Novosibirsk, Russia; timazharkov74@gmail.com (T.D.Z.); mironovaforwork@gmail.com (E.M.M.); markov_ov@niboch.nsc.ru (O.V.M.); jsvbsasp@yandex.ru (S.A.Z.); svetakh@niboch.nsc.ru (S.N.K.)

**Keywords:** lipophilic oligonucleotides, phosphoryl guanidine, triazinyl phosphoramidate, conjugates, delivery

## Abstract

Lipophilic oligonucleotide conjugates represent a powerful tool for nucleic acid cellular delivery, and many methods for their synthesis have been developed over the past few decades. In the present study, a number of chemical approaches for the synthesis of different fork- and comb-like dodecyl-containing oligonucleotide structures were performed, including use of non-nucleotide units and different types of phosphate modifications such as alkyl phosphoramidate, phosphoryl guanidine, and triazinyl phosphoramidate. The influence of the number of introduced lipophilic residues, their mutual arrangement, and the type of formed modification backbone on cell penetration was evaluated. The results obtained indicate great potential in the developed chemical approaches, not only for the synthesis of complex oligonucleotide structures but also for the fine-tuning of their properties.

## 1. Introduction

Modified oligonucleotides are widely used as a convenient instrument in numerous scientific fields including medicine [1,2,3]. While being applied as therapeutics, oligonucleotides contain chemical modifications in their structure that enhance several crucial properties [4,5,6]. One of them is the oligonucleotide cellular uptake and its further intracellular accumulation.

The most common approach to enhance the efficiency of oligonucleotide transfection is the introduction of various hydrophobic groups into their structure. There are many different examples of lipophilic oligonucleotide conjugates containing cholesterol, tocopherol, fatty acids, and long-chain aliphatic groups, that demonstrate improved cellular penetration and biological potency [7,8,9,10,11,12,13,14,15,16,17,18].

Apart from the nature of the introduced lipophilic group, several factors influence the efficiency of the uptake of NA derivatives by cells. These factors include the number of lipophilic groups, their relative positions, and the chemical structure of the modification unit that bears them [19,20,21]. Varying different structural parameters, while keeping the nature of the introduced lipophilic group constant, allows for the assessment of the factors’ contribution to the intracellular accumulation efficacy of such oligonucleotide derivatives [22,23].

In the present study, the dodecyl moiety was chosen as the lipophilic group to be incorporated. In contrast to lipophilic groups widely used for delivery, such as cholesterol residues, the dodecyl moiety possesses “moderate” hydrophobicity, which allows one to estimate the cumulative effect of improved intracellular accumulation within an increasing number of dodecyl groups introduced into oligonucleotide structure. Previously, comb-like dodecyl-containing NA constructions with phosphoramidates or non-nucleotide modifications bearing one to three dodecyl residues were synthesized and their delivery efficiency was evaluated [19]. Regardless of the modification methods used, the levels of intracellular accumulation were shown to increase exponentially with an increasing number of dodecyl residues.

Herein, we extended dodecyl insertion methods using novel types of both non-nucleotide units and phosphate modifications, and synthesized comb- and fork-like structures [24] with multiple dodecyl residues to evaluate the influence of the mutual arrangement of lipophilic groups and backbone-type modification on the transfection efficiency of lipophilic oligonucleotide derivatives.

## 2. Results

There are two steps in the protocol of the solid-phase phosphoramidite oligonucleotide synthesis cycle, which are often used to introduce modifications. The first option is based on the use of different phosphoramidite monomers and modifiers during the condensation step of the regular synthetic cycle protocol, whereas the second option involves the application of an alternative oxidation step. In our previous study, we have already demonstrated the possibility of implementing both variants for the introduction of lipophilic groups by synthesizing the set of oligonucleotides using non-nucleotide dodecyl monomers and oxidative amination in the presence of dodecylamine [19]. In terms of the relative position of dodecyl residues, these oligonucleotides represent comb-like structures with a sequential localization of hydrophobic moieties, as opposed to fork-like structures with a parallel localization of modification groups [24]. In the present study, we implemented new variants of both approaches to introduce modifications in oligonucleotides with multiple dodecyl residues with comb- and fork-like structure types.

### 2.1. Synthesis of Dodecyl-Containing Oligonucleotides Utilizing Non-Nucleotide Phosphoramidites

**Figure 1:**
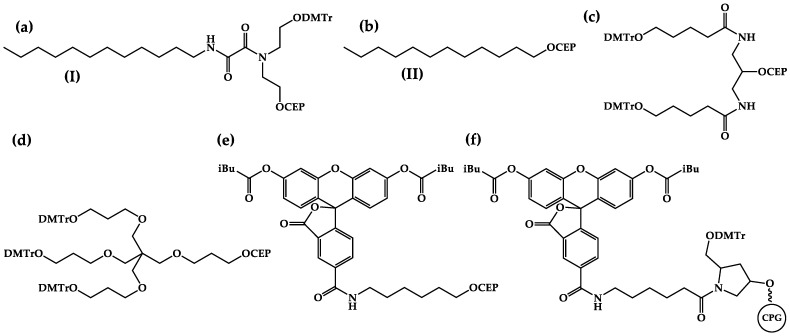
Structures of non-nucleotide monomers (**a**,**c**,**d**), 5′-modifiers (**b**,**e**), and solid support (**f**) used in this study. DMTr—4,4′-dimethoxytrityl; CEP—(2-cyanoethyl-N,N-diisopropyl)phosphoramidite; iBu—isobutyryl; CPG—controlled pore glass.

The developed dodecyl-containing monomer (I) on the rigid oxalamide backbone [25] (Figure 1a) was used to obtain the oligonucleotide N3 (Table 1), which bears three sequentially introduced dodecyl-containing non-nucleotide units at the 3′-region as a comb-like structure with proven cellular delivery efficiency [19].

A typical 5′-modifier (II) (Figure 1b), which is the simplest structural variant of a dodecyl-containing phosphoramidite, was obtained in one step from dodecanol-1 by a standard phosphitylation reaction. The introduction of only one dodecyl moiety into the oligonucleotide structure is not sufficient to provide efficient intracellular accumulation despite its relative hydrophobicity [19]; therefore, such a 5′-modifier is not represented in popular manufacturers’ catalogs. In order to perform the multiple incorporation of this modifier, commercially available doubler (Figure 1c) and trebler (Figure 1d) monomers were used. Applying a combination of such phosphoramidites, oligonucleotides O1–O3 with a fork-like modification structure bearing one to three dodecyl residues at the 5′-region were obtained (Table 1).

Modifications were introduced into a fluorescein-labeled oligonucleotide with a randomized sequence without any known specific molecular target: 5′-CTG-ACT-ATG-AAG-TATT-3′, which was previously used in intracellular delivery study [26]. Oligonucleotides O1–O3 and N3 were synthesized using the corresponding FAM modifiers at the 3′ end (Figure 1f) and the 5′ ends (Figure 1e), respectively, as well as the control oligonucleotide C (see Table 1).

When evaluating the delivery efficiency, to avoid the unwanted influence of the hydrophobic environment on the fluorescence efficiency, lipophilic groups and fluorescein residue were introduced at the opposite ends of oligonucleotide sequence in all dodecyl-containing structures [27,28].

### 2.2. Synthesis of Dodecyl-Containing Oligonucleotides with Modified Phosphate Group

Another variant of modification introduction is based on the alteration of the oxidation step during automated phosphoramidite solid-phase synthesis. In the standard protocol, after the addition of a regular monomer unit to the growing oligonucleotide chain, a phosphite triester intermediate is formed, which is then oxidized to the phosphate form by iodine in THF/pyridine solution treatment in the presence of water. Replacing the standard oxidation mixture with alternative reagents allows for the obtainment of various phosphate-modified oligonucleotide derivatives.

**Figure 2:**
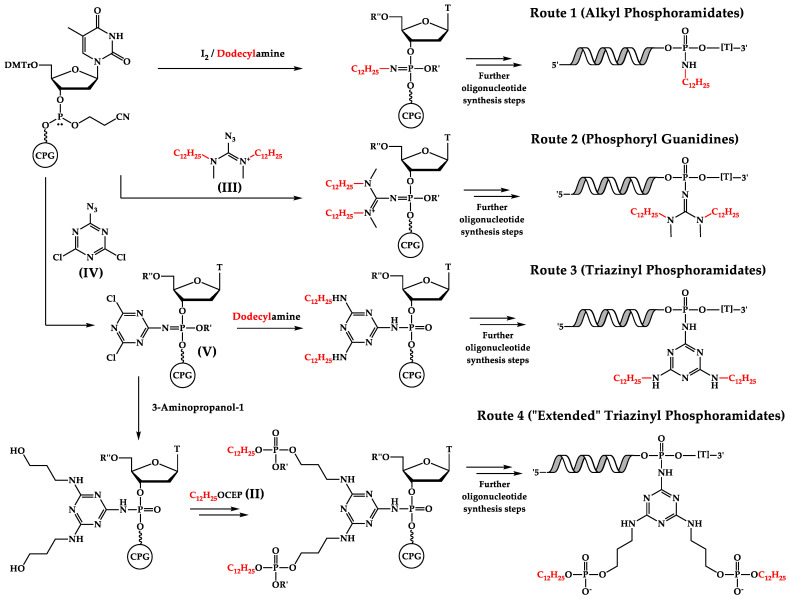
Various routes for the synthesis of phosphate-modified oligonucleotide derivatives—alkyl phosphoramidates (route 1), phosphoryl guanidines (route 2), triazinyl phosphoramidates (route 3), and “extended” triazinyl phosphoramidates (route 4). R″ = DMTr; R′ = 2-cyanoethyl. DMTr—4,4′-dimethoxytrityl; CEP—(2-cyanoethyl-N,N-diispropyl)phosphoramidite.

One of the earliest approaches to the synthesis of phosphate-modified NA derivatives within alternative oxidation was based on oxidative amination [29]. By changing water to an appropriate primary amine in oxidation mixture, alkyl phosphoramidate oligonucleotides can be synthesized. Particularly, the synthesis of dodecyl phosphoramidate derivatives through the treatment of the phosphite triester unit with iodine in a pyridine/DMF solution in the presence of dodecylamine has already been shown [19]. The developed protocol was used for the synthesis of alkyl phosphoramidate derivative N2 containing dodecyl residues at two neighboring phosphate groups in the 3′ end region of the oligonucleotide sequence as a comb-like structure with proven cellular delivery efficiency [19] (Figure 2, Route 1).

Besides the use of oxidative amination with dodecylamine to obtain phosphate-modified oligonucleotides with two dodecyl residues, other variants of the alternative oxidation step have also been applied. One such variant is based on the Staudinger reaction, where the oxidation of a phosphite triester intermediate occurs via interaction with various organic azides. The use of alkyl azides was previously shown to lead to phosphoramidate formation under much harsher conditions compared to applying appropriate alkyl amines in oxidative amination [29]. On the contrary, different electron-deficient azides possess high reactivity in the Staudinger reaction and their application has recently been adopted for effectively obtaining the corresponding NA derivatives [30]. For instance, applying cationic tetrasubstituted diaminocarbenium azides allows one to obtain different phosphoryl guanidine oligonucleotide derivatives [31].

In this study, the phosphoryl guanidine derivative GN2 containing two dodecyl residues, and at the same modifying the phosphate group in the 3′ end region of the oligonucleotide sequence (Table 2), was synthesized (Figure 2, Route 2) using N,N′-dimethyl-N,N′-didodecyldiaminocarbenium azide (III, Figure 2) according to the described protocol [31].

A more complex variant of the alternative oxidation step was carried out with 2-azido-4,6-dichloro-1,3,5-triazine (IV, Figure 2) [26]. Here, the initial Staudinger reaction with organic azide led to a triazine-containing intermediate with two reactive centers in the oligonucleotide structure (V, Figure 2). Subsequent treatment with dodecylamine resulted in the introduction of the desired functional residues into the triazine backbone. Following further standard automated synthetic steps (Figure 2, Route 3), the triazinyl phosphoramidate TN2 bearing two dodecyl moieties was obtained.

We also tested one possible combination of both amidite modifiers and alternative oxidation approaches for the synthesis of an oligonucleotide with two dodecyl residues. After the obtaining the Staudinger reaction intermediate (V), instead of dodecylamine for the direct incorporation of dodecyl residues, 3-aminopropanol-1 was used to introduce two additional unprotected primary hydroxy groups into the triazine backbone. After the introduction of dodecyl units applying the synthesized 5′-modifier (Figure 1b), oligonucleotide synthesis was continued (Figure 2, Route 4) to obtain an alternative triazinyl phosphoramidate, TO2, bearing two dodecyl moieties connected to the triazine backbone by additional linker arms (Table 2).

This combined approach was successfully performed on a model octathymidylate sequence. However, while attempting to obtain the target heteronucleotide derivative, a significant decrease in the yield of the desired product occurred. Presumably, this is related to the steric hindrance of substituents in the triazine backbone, which negatively affects the further condensation stages of nucleotide units. Considering this, we decided to use the obtained modified octathymidylate TO2 for the initial evaluation of the influence of this modification on intracellular accumulation.

Thereby, a number of different classes of oligonucleotides with comb- and fork-like structures bearing two dodecyl residues, namely alkyl phosphoramidate (N2), phosphoryl guanidine (GN2), and triazinyl phosphoramidate with (TO2) or without (TN2) additional linker arms, were obtained using various alternative oxidation steps.

All oligonucleotides were purified using reversed-phase HPLC and identified using ESI mass spectrometry. The HPLC profiles of the reaction mixtures of the modified oligonucleotides (Figure S1), the data of the mass spectrometry analysis (Figure S2), and the results of the thermal denaturation experiment of the studied oligonucleotides with a complementary unmodified oligonucleotide (Table S1) are provided in “Supplementary Materials”.

### 2.3. Evaluation of the Efficiency of the Intracellular Accumulation of Dodecyl-Containing Fork- and Comb-like Oligonucleotide Derivatives

**Figure 3:**
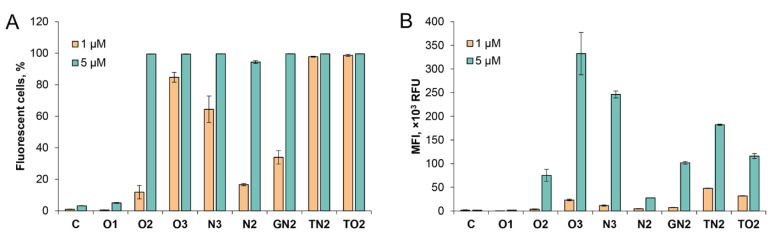
Intracellular accumulation of FAM-labeled dodecyl-containing oligonucleotides into HEK293T cells at two concentrations: orange bars—1 µM; green bars—5 µM. The percentage of fluorescent cells (**A**) and the mean fluorescence intensity (MFI) (**B**) were measured using flow cytometry 4 h post transfection. Data are presented as MEAN ± SD. All experimental points were run in triplicate for statistical analysis.

In order to evaluate the influence of factors such as the number of lipophilic groups and their relative position to each other on transfection efficiency, experiments on the intracellular delivery of the obtained dodecyl-containing oligonucleotide derivatives were performed on the HEK293T cell line. Human embryonic kidney cells have been used as a common cell line in studies of nucleic acid delivery approaches with various transfectants [32,33,34], including previously investigated dodecyl-containing oligonucleotide derivatives [19]. Dodecyl-containing oligonucleotides, as well as a nonmodified oligonucleotide without lipophilic groups (oligonucleotide C) serving as a negative control, were incubated with HEK293T cells in serum-free culture media for 4 h. The intracellular accumulation of the oligonucleotides was analyzed using flow cytometry (Figure 3) and confocal microscopy (Figure S3 in Supplementary Materials). The transfection efficiency was assessed using two parameters—the percentage of fluorescent cells and the mean fluorescence intensity.

Initially, dodecyl-containing oligonucleotides were delivered to the cells at a concentration of 1 µM, which was used in our previous work [19]. However, due to the diverse percentage of transfected cells for different dodecyl-containing oligonucleotide derivatives, the concentration of the oligonucleotides was increased to 5 µM in order to properly analyze the transfection efficiency of the oligonucleotides under relatively similar conditions. It was demonstrated that all oligonucleotides containing at least two dodecyl residues were accumulated in approximately 100% of the cells at this concentration (Figure 3A). Therefore, the relative efficiency of oligonucleotide delivery was further evaluated in a comparative manner by the level of mean fluorescence intensity at a concentration of 5 µM (Figure 3B).

It was clearly shown that the transfected oligonucleotides at both concentrations demonstrated a common tendency for intracellular accumulation (Figure 3B). Derivative O1 bearing one 5′-dodecyl residue was barely detectable in the cells—the levels of the number of fluorescent cells and fluorescence intensity were comparable to the unmodified control oligonucleotide C (Figure 3B). At the same time, a sequential increase in the number of introduced 5′-dodecyl residues with a fork-like arrangement type (oligonucleotides O2 and O3) resulted in an exponential growth of the fluorescence intensity of transfected cells. These data are in good agreement with the previously published results of a similar experiment for a series of oligonucleotides, where the introduction of dodecyl residues was carried out using both non-nucleotide dodecyl monomers (comb-like, non-nucleotide series) and a dodecylamine on the oxidative amination step of oligonucleotide synthesis (comb-like, phosphoramidate series) [19]. Both series showed similar delivery efficiency for oligonucleotides with the same number of dodecyl residues. In the present study, the oligonucleotides N2 (phosphoramidate) and N3 (non-nucleotide) were obtained as analogues of two- and three-dodecyl comb-like modified oligonucleotides with proven cellular delivery efficiency [19].

It should be noted that the transfection efficiency of O2 and O3 oligonucleotides exceeds that of N2 and N3 oligonucleotides. It is possible that the fork-like arrangement of dodecyl groups in O-series oligonucleotides is more flexible and thus more preferable for cumulative hydrophobic interactions than N-series oligonucleotides with a rigid comb-like arrangement type modification structure.

After defining the tendency of intracellular accumulation’s exponential growth with the number of lipophilic groups, we decided to compare all derivatives bearing two dodecyl moieties to estimate the modification backbone’s impact. Among such oligonucleotides, alkyl phosphoramidate comb-like derivative N2 with dodecyl residues at two neighboring phosphate groups showed the lowest transfection potential. On the contrary, oligonucleotides with the fork-like type localization carrying two dodecyl residues within the same phosphate group (GN2, TN2, and TO2 oligonucleotides) or two 5′-dodecyl residues (oligonucleotide O2) were more efficiently accumulated in the cells (Figure 3).

Oligonucleotides O2 and TO2, both with dodecyl residues introduced using flexible linkers (see Table 1 for the O2 structure and Table 2 for the TO2 structure), demonstrated comparable delivery efficiency despite having different oligonucleotide sequences. In contrast, oligonucleotides GN2 and TN2, both with rigid and spatially similar backbone types, demonstrated a reliable distinction in intracellular accumulation. Presumably, triazine as a part of phosphate modification (TN2) provided an additional contribution to cellular uptake unlike the guanidine residue (GN2).

Thus, it can be concluded that not only the number of introduced hydrophobic groups, but also the arrangement pattern as well as the type of modification backbone, can significantly affect the transfection efficiency of lipophilic NA derivatives.

## 3. Discussion

In this work, a series of dodecyl-containing oligonucleotides were obtained using various synthetic approaches. Several dodecyl residues were introduced into the oligonucleotide chain using non-nucleotide units and different types of phosphate modifications—alkyl phosphoramidate, phosphoryl guanidine, and triazinyl phosphoramidates. The influence of factors such as the number of introduced lipophilic residues, their mutual arrangement, and the type of formed modification backbone on cellular delivery was evaluated.

Nowadays, the application of phosphoramidite monomers, 5’-modifiers, and modified CPG supports is a classic approach for introducing various non-nucleotide units into the oligonucleotide structure on DNA/RNA synthesizers and has become a routine procedure. A wide range of non-nucleotide phosphoramidites and solid supports can be found in the catalogs of many popular suppliers of reagents for oligonucleotide synthesis. Complex constructions can be made using combinations of different types of non-nucleotide units during automated synthesis. In addition to the oligonucleotide (N3) obtained through sequential multiple introductions of previously investigated non-nucleotide dodecyl monomers [19], in this study, we used the obtained 5’-dodecyl modifier together with commercially available doubler (O2) and trebler (O3) monomers to create novel lipophilic NA constructions.

After the discovery of a new class of phosphate-modified NA derivatives, phosphoryl guanidines [35], and the subsequent development of a highly efficient method for the preparation of various phosphate derivatives on modern DNA/RNA synthesizers [30], which involves the use of highly reactive electron-deficient azides in the Staudinger reaction, it can be stated that the phosphate modification chemistry of nucleic acids is entering a new stage of technological development. In addition to the widely used phosphorothioate modification [36], the researcher’s toolkit now includes new promising classes of phosphate modifications such as phosphoryl guanidines [35,37,38] and sulfonyl phosphoramidates [39]. It was shown that the introduction of several phosphoryl guanidine modifications can improve the potency, distribution, and durability of corresponding therapeutic phosphorothioate oligonucleotides with different types of biological action [40,41,42], and sulfonyl phosphoramidate modification showed some promising results in improving the therapeutic index of antisense oligonucleotides [43].

The alternative oxidation step of oligonucleotide synthesis can serve not only as a source for changing the nature of the phosphate part of the oligonucleotide backbone, but also allows one to introduce a wide range of substituents. Here, we exploited our developed strategy of using electron-deficient azides in the Staudinger reaction as a useful conjugation method in addition to the previously studied oxidative amination approach (N2) [19]. Thus, N,N′-dimethyl-N,N′-didodecyldiaminocarbenium azide was used for the direct introduction of desired dodecyl moieties into the oligonucleotide structure (GN2), whereas the use of 2-azido-4,6-dichloro-1,3,5-triazine led to the formation of a reactive center in the oligonucleotide structure for the introduction of desired moieties during treatment with appropriate amines (TN2 and TO2).

It is worth noting that use of phosphoramidite monomers and different types of alternative oxidative steps as a source of introducing the desired modifications are applied on independent parts of the oligonucleotide synthetic cycle, and in this way, it could be combined with the synthesis of the same oligonucleotide.

A number of oligonucleotide derivatives containing long alkyl chains (C12–C28) have been described and investigated for cell delivery [19,26,44,45,46,47]. It was demonstrated that such lipophilic conjugates, including a previously studied dodecyl-containing oligonucleotide, not only reliably accumulated inside the cells without transfection agents [19,44,47], but also possessed low cytotoxicity [26,44,45,46]. As expected, the efficiency of the intracellular accumulation of our novel series of dodecyl-conjugated oligonucleotides was also primarily affected by the number of introduced dodecyl groups, and the increase in cellular uptake was exponential [19]. An interesting finding of the present study is the alteration of the cellular accumulation of oligonucleotides with the same number of dodecyl groups but different modification structures. Thus, oligonucleotide GN2 with dodecyl residues on a rigid phosphoryl guanidine backbone showed comparable results to oligonucleotide O2 with residues on flexible linkers, but notably lower accumulation than TN2 with a rigid and spatially similar triazinyl phosphoramidate backbone.

To sum up, with the example of the synthesis of different fork- and comb-like oligonucleotide structures with multiple dodecyl residues, we demonstrated that existing approaches such as the use of non-nucleotide phosphoramidites together with recently developed phosphate modification methods can not only be a powerful tool for the synthesis of various oligonucleotide derivatives, but they also have great potential for fine-tuning their properties.

## 4. Materials and Methods

### 4.1. Oligonucleotide Synthesis

The synthesis of dodecyl phosphoramidite monomer (I) was performed as described in [25].

The synthesis of dodecyl phosphoramidite 5′-modifier (II):

A mixture of 5-(ethylthio)-1H-tetrazole (0.53 g, 4 mmol, 1 eq.), 2-cyanoethyl-bis(N,N-diisopropylamine)phosphite (1.31 mL, 4 mmol, 1 eq.), and DIPEA (1.62 mL, 9.2 mmol, 2.3 eq.) was dissolved in 7.5 mL of dry acetonitrile and stirred at room temperature for 30 min. The resulting solution was added dropwise to dodecanol-1 (0.41 mL, 4 mmol, 1 eq.); then, the reaction mixture was stirred at room temperature for 1 h. Then, ¾ of the volume of the solution was evaporated in a vacuum. Dichloromethane (150 mL) was added and then the solution was washed with 0.3 M KH_2_PO_4_ (4 × 50 mL). The organic layer was dried over anhydrous Na_2_SO_4_ and evaporated; the product was obtained as a white-yellow oil with 98% yield (1.02 g). Rf = 0.85, TLC system—hexane/EtOAc, 9:1. ^1^H NMR (DMSO-d6, 80 MHz, 20 °C): d3.90 (2H, m, OCH_2_CH_2_CN), 3.41 (4H, complex signal, N[CH(CH_3_)_2_]_2_,OCH_2_CH_2_(CH_2_)_9_CH_3_), 2.63 (2H, t, OCH_2_CH_2_CN), 1.56 (2H, m, OCH_2_CH_2_(CH_2_)_9_CH_3_), 1.34–1.12 (30H, m, N[CH(CH_3_)_2_]_2_, OCH_2_CH_2_(CH_2_)_9_CH_3_, 0.87 (3H, t).

The synthesis of N,N′-dimethyl-N,N′-didodecyldiaminocarbenium azide (III) was performed as described in [31].

The synthesis of 2-azido-4,6-dichloro-1,3,5-triazine (IV) was performed as described in [26].

The standard phosphoramidite solid-phase synthesis of all modified and unmodified oligonucleotides containing phosphodiester linkages was carried out on an ASM-800 DNA/RNA synthesizer (Biosset, Novosibirsk, Russia). Oligonucleotides were synthesized at the 0.4 µmol scale, using standard commercial 2-cyanoethyl deoxynucleoside phosphoramidites and CPG solid supports (Glen Research, San Diego, CA, USA).

Oligonucleotide N3 with dodecyl-containing non-nucleotide units was synthesized using a dodecyl phosphoramidite monomer (I) as described in [19].

Oligonucleotides O1, O2, and O3 containing 1 to 3 dodecyl moieties at the 5′ end region were synthesized using a dodecyl phosphoramidite (I) 0.1 M solution in anhydrous acetonitrile with an extended coupling time of 45 min and commercially available doubler and trebler phosphoramidites (Glen Research, San Diego, CA, USA), according to the manufacturer’s protocols for the modification introduction step.

Oligonucleotide N2 with two dodecyl-containing phosphoramidate modifications was synthesized using dodecylamine during the modified oxidation step as described in [19].

Oligonucleotide GN2 containing a phosphoryl guanidine modification bearing two dodecyl moieties was synthesized using organic azide (III) during the modified oxidation step as described in [31].

Oligonucleotide TN2 containing a triazinyl phosphoramidate modification bearing two dodecyl moieties was synthesized using organic azide (IV) during the modified oxidation step as described in [26].

Oligonucleotide TO2 containing an extended triazinyl phosphoramidate modification bearing two dodecyl moieties on additional linker arms was synthesized using organic azide (IV) during the modified oxidation step as described in [26], with the following distinction: 10% anhydrous acetonitrile solution of 3-aminopropanol-1 (90 min, T = 55 °C) was used instead of dodecylamine solution after obtaining the reactive intermediate (V). The synthesized dodecyl phosphoramidite (I) was used to introduce dodecyl residues as described above.

For the introduction of the 6-carboxyfluoresceine (FAM) residue either at the 5′ end or 3′ end of the oligonucleotide sequence, the corresponding phosphoramidite or modified CPG (Lumiprobe, Moscow, Russia) was used according to the manufacturer’s protocols. The CPG cleavage of FAM-containing oligonucleotides was first performed in aqueous ammonia (30% *m/v*, 15 min, 55 °C), and then aqueous methylamine (40% *m/v*, 15 min, 55 °C) was added equally to complete the process.

### 4.2. Oligonucleotide Purification and Identification

A quality assessment of oligonucleotide synthesis was performed through reversed-phase HPLC analysis on a Millichrom A02 system using a ProntoSIL-120-5-C18 column 2 × 75 mm (Econova, Novosibirsk, Russia) in a linear gradient of acetonitrile 0–50% or 0–90% in 20 mM triethylammonium acetate, pH 7.0, at a flow rate of 200 μL/min, and with detection on 260, 280, and 300 nm wavelengths.

Modified oligonucleotides were isolated through reversed-phase HPLC on an Agilent1200 HPLC system (Santa Clara, CA, USA) using a Zorbax SB-C18 5 mm column 4.6 × 150 mm in a linear gradient of acetonitrile 0–50% or 0–90% in 20 mM triethylammonium acetate, pH 7.0, at a flow rate of 2 mL/min, and with detection on 260, 280, 300, and 500 nm wavelengths. Fractions containing the desired product were collected, evaporated several times with a 1:1 EtOH/H_2_O mixture, concentrated in vacuo, and precipitated by 1 mL of 2% LiClO_4_ in acetone. After centrifugation at 14,500 rpm for 2 min, washing with acetone, and drying in air for 20 min at 40 °C, the oligonucleotide precipitates were dissolved in 0.1 mL of deionized water and stored at −20 °C.

Molecular masses of oligonucleotides were confirmed by LC-MS/MS ESI-MS on an Agilent G6410A mass spectrometer (Santa Clara, CA, USA) in a negative-ion detection mode. The samples were prepared by dissolving oligonucleotides in 20 mM triethylammonium acetate in 60% aq. acetonitrile at a concentration of 0.1 mM in 10 μL of the sample. Analysis was carried out using 80% aq. acetonitrile as an eluent at a flow rate of 0.1 mL/min and using standard device settings. Molecular masses were calculated from the experimental m/z values obtained for each sample.

### 4.3. Analysis of Intracellular Accumulation of Oligonucleotides

HEK293T (human embryonic kidney) cells were cultivated in DMEM medium (Sigma-Aldrich, St. Louis, MO, USA) supplemented with 10% fetal bovine serum ( HyClone, GE Healthcare, Chicago, IL, USA) and 1% antibiotic/antimycotic solution (100 units/mL of penicillin, 0.1 mg/mL of streptomycin, and 0.25 g/mL of amphotericin) ( MP Biomedicals, Santa Ana, CA, USA) at 37 °C in a humidified atmosphere with 5% CO_2_ (hereinafter, standard conditions).

For the transfection experiments, HEK293T cells were seeded in 24-well plates at a density of 150 × 10^3^ cells/well in 500 µL of complete DMEM medium and cultivated to adhere to standard conditions overnight. The next day, the cultivation medium was replaced with a serum- and antibiotic-free DMEM medium supplemented with 1 or 5 µM of the oligonucleotides under study, and the cells were transfected for 4 h in standard conditions. The cells were detached from the plates with TrypLE Express Enzyme (Gibco, Grand Island, NY, USA), resuspended in complete DMEM medium to inactivate the enzyme, washed in PBS, and fixed in 2% formaldehyde in PBS. The intracellular accumulation of the oligonucleotides was examined through flow cytometry using a NovoCyte flow cytometer (ACEA Biosciences, Santa Clara, CA, USA). Flow cytometry data were processed with NovoExpress software v. 1.1.0 (ACEA Biosciences, Santa Clara, CA, USA). All experiments were run in triplicate for statistical analysis. Transfection efficiency was characterized by the percentage of fluorescent-positive cells and the mean fluorescence intensity (MFI) of cells in a sample.

### 4.4. Statistical Analysis

Data were statistically processed using one-way ANOVA with Tukey post hoc testing. *p* < 0.05 was considered to be significant. GraphPad Prism v. 8.0.1 software (GraphPad Software Inc., San Diego, CA, USA) was used for statistical analysis.

## Figures and Tables

**Table 1:** List of oligonucleotides synthesized using dodecyl-containing phosphoramidites and structures of introduced lipophilic residues. Non-nucleotide and fluorescence units in the oligonucleotide sequence which were incorporated using the corresponding phosphoramidites or CPG: [Dcyl]—5′-terminal dodecyl moiety; [X]—dodecyl unit; [Db]—doubler; [Tb]—trebler; [FAM]—5′-fluorescein moiety; [FAM’]—3′-fluorescein moiety.

Code	Sequence (5′–3′)	MS (Calc/Found)
O1	[Dcyl]-CTG-ACT-ATG-AAG-TATT-[FAM’]	5794.1/5796.0
O2	[Dcyl]_2_[Db]-CTG-ACT-ATG-AAG-TATT-[FAM’]	6394.7/6392.4
O3	[Dcyl]_3_[Tb]-CTG-ACT-ATG-AAG-TATT-[FAM’]	6721.1/6722.8
N3	[FAM]-CTG-ACT-ATG-AAG-TAT-[X][X][X]-T	6706.0/6705.6
C	[FAM]-CTG-ACT-ATG-AAG-TATT	-/-
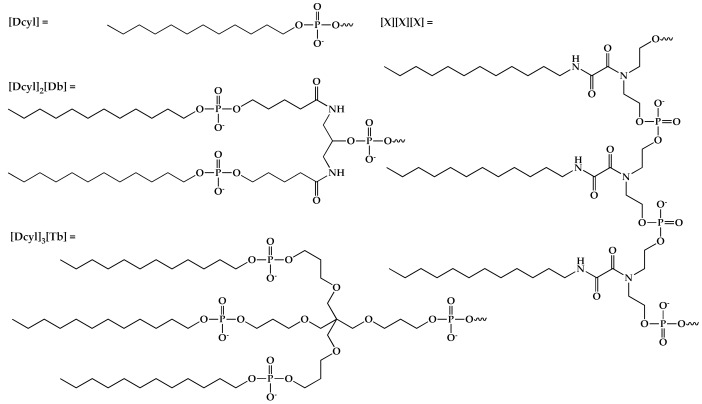		

**Table 2:** List of phosphate-modified dodecyl-containing oligonucleotides obtained using the alternative oxidation approach. [FAM]—5′-fluorescein moiety. *—modified phosphate group position.

Code	Sequence (5′–3′)	MS (Calc/Found)
N2	[FAM]-TTT-CTG-ACT-ATG-TA*^a^T*^a^T	5724.3/5731.8
GN2	[FAM]-CTG-ACT-ATG-AAG-TAT*^b^T	5838.4/5837.4
TN2	[FAM]-CTG-ACT-ATG-AAG-TAT*^c^T	5877.4/5877.4
TO2	[FAM]-TTT-TTT-T*^d^T	3629.9/3628.8
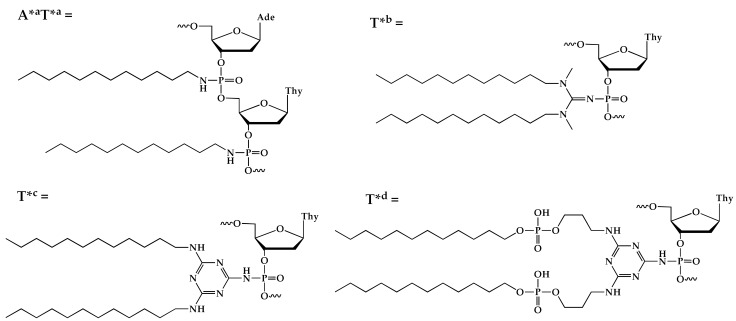		

## Data Availability

All data can be easily obtained and linked in the respective sections.

## Supplementary Materials

The following supporting information can be downloaded at: https://www.mdpi.com/article/10.3390/ijms241914637/s1.
