# Supplementary material for: Fork- and Comb-like Lipophilic Structures: Different Chemical Approaches to the Synthesis of Oligonucleotides with Multiple Dodecyl Residues
Source: Int J Mol Sci. 2023 Sep 27;24(19):14637. doi: 10.3390/ijms241914637 (PMC10572690; doi:10.3390/ijms241914637)
Supplement: Supplementary file 1 [file ijms-24-14637-s001.zip › ijms-2601175-supplementary.pdf]

SUPPLEMENTARY MATERIAL S1

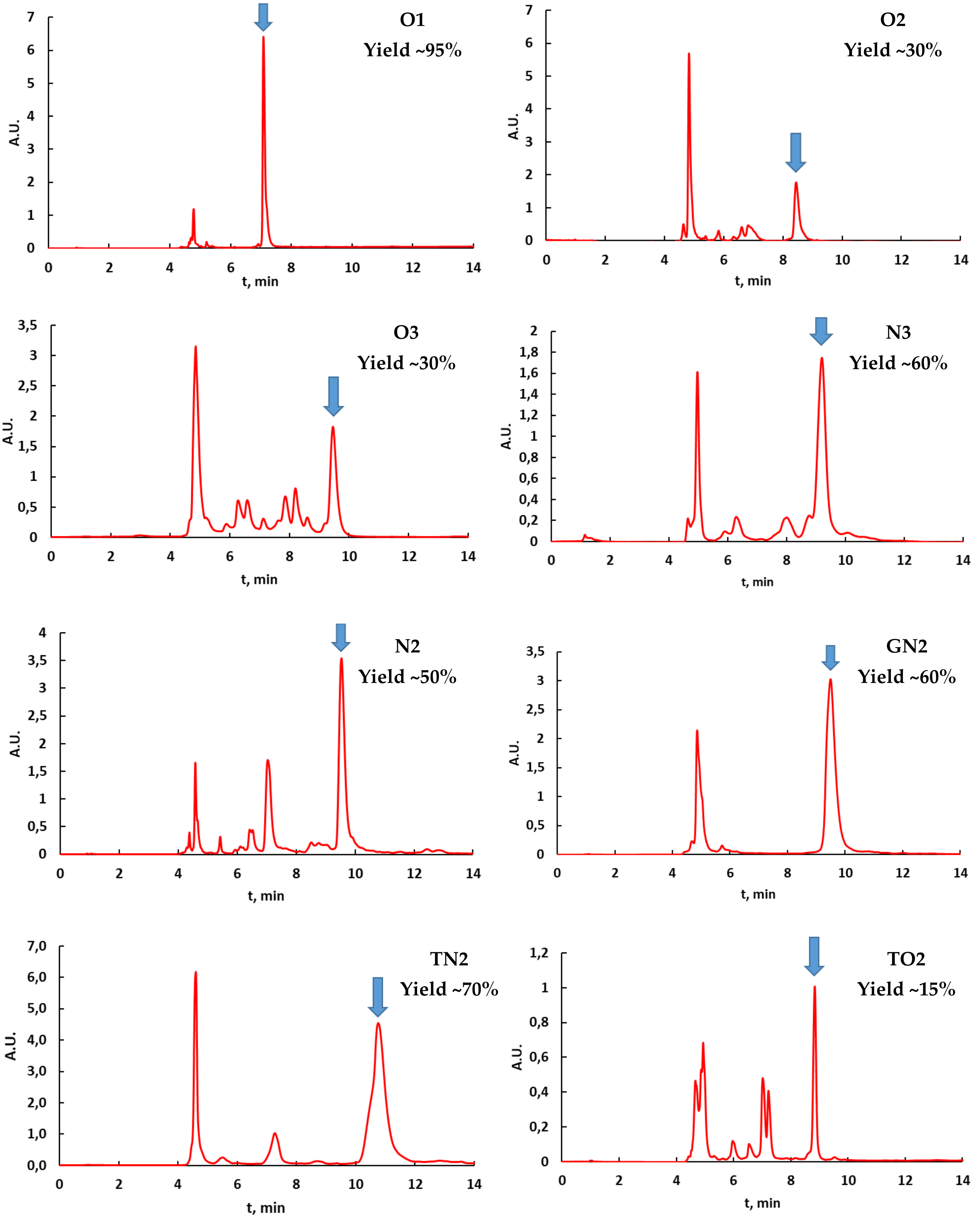

Figure S1. Analytical RP HPCL profiles of reaction mixture of synthesis of corresponding modifed oligonucleotides. Blue arrow shows peak that was collected and identified as desired product. Yields were calculated as the ratio of the peak area of the desired product to the total absorption at the wavelength 260 nm.

SUPPLEMENTARY MATERIAL S2

O1 5′- [Dcyl]-CTG-ACT-ATG-AAG-TATT-[FAM] -3′

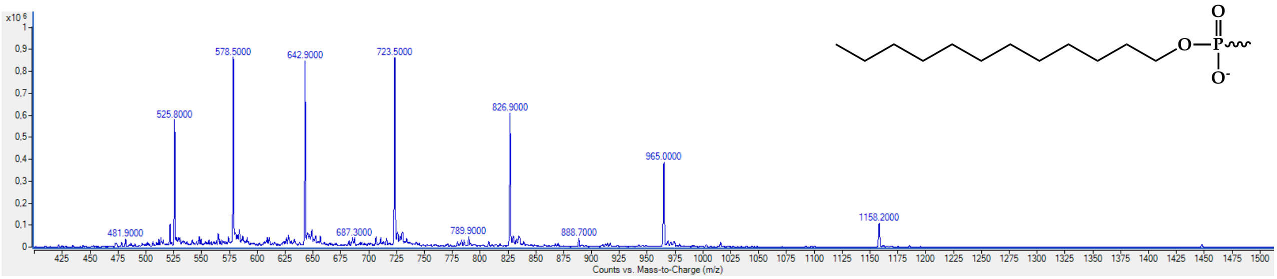

O2 5′- [Dcyl]$_2$[Db]-CTG-ACT-ATG-AAG-TATT-[FAM] -3′

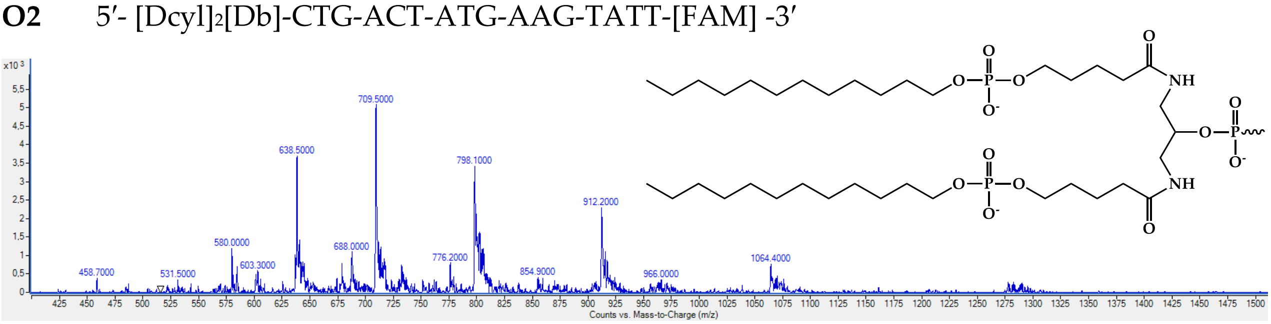

O3 5′- [Dcyl]$_3$[Tb]-CTG-ACT-ATG-AAG-TATT-[FAM] -3′

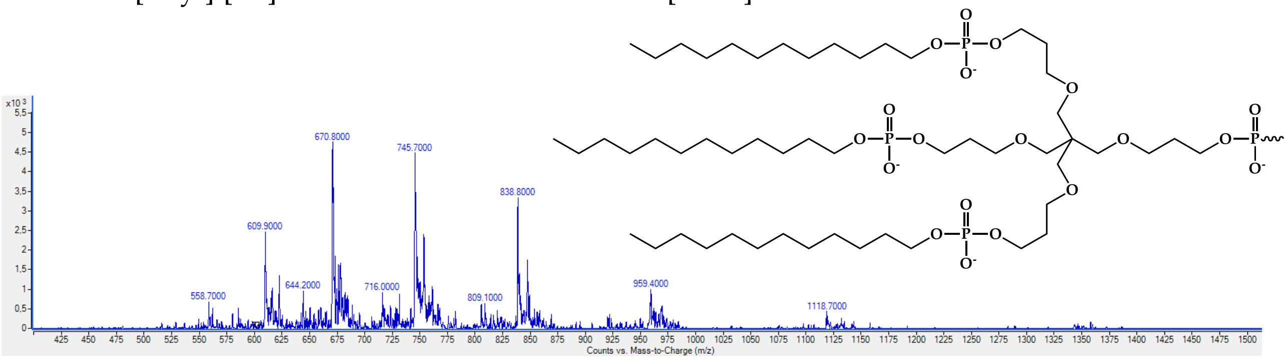

X3 5′- [FAM]-CTG-ACT-ATG-AAG-TAT-[X][X][X]-T -3′

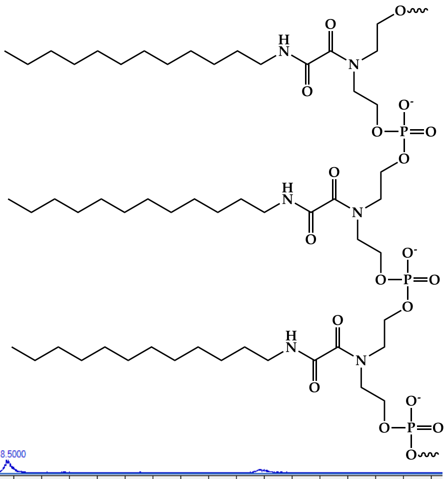

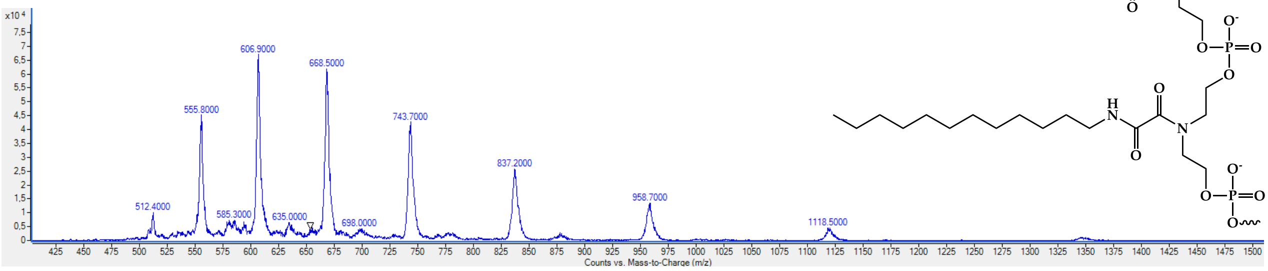

SUPPLEMENTARY MATERIAL S2 (Continued)

N2 5′- [FAM]-CTG-ACT-ATG-AAG-TA*T*T -3′

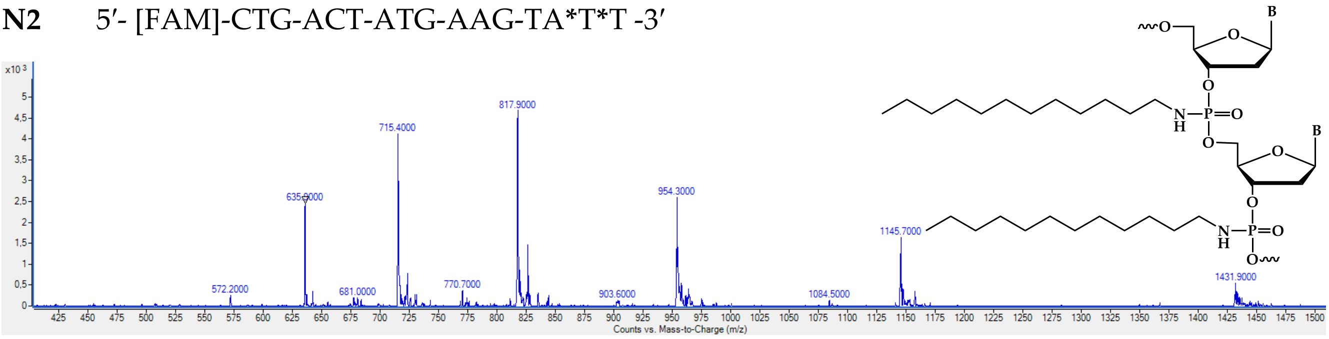

GN2 5′- [FAM]-CTG-ACT-ATG-AAG-TAT*T -3′ (Reaction mixture)

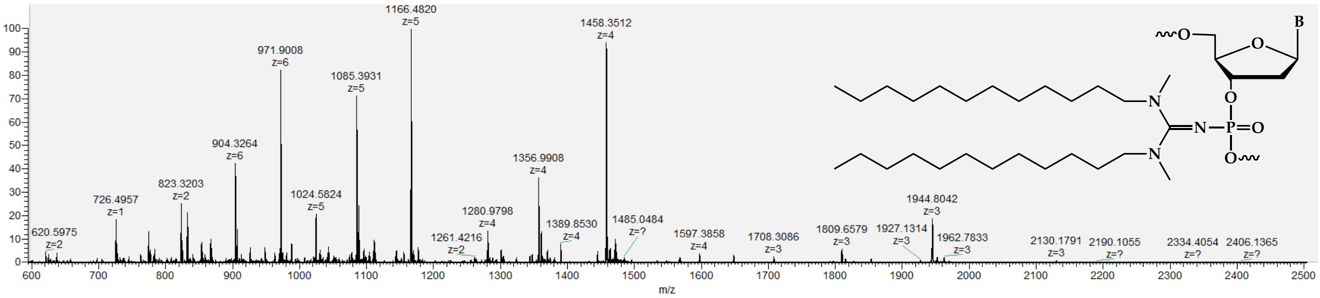

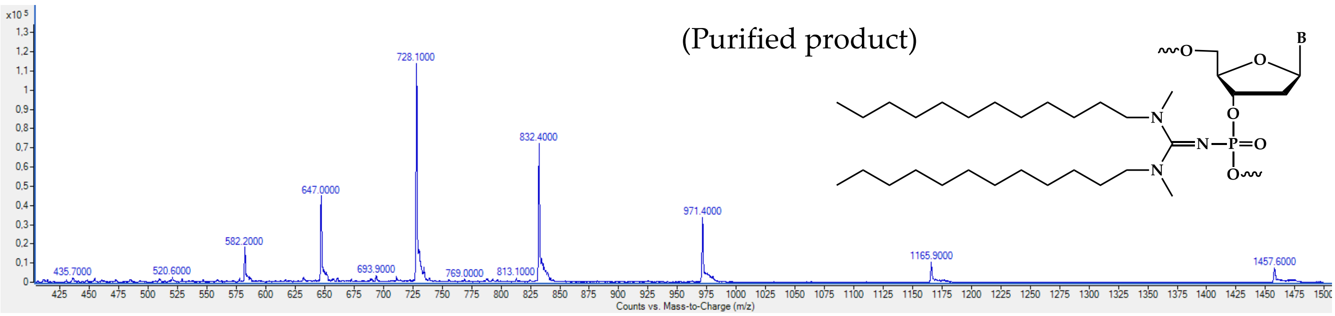

TN2 5′- [FAM]-CTG-ACT-ATG-AAG-TAT*T -3′

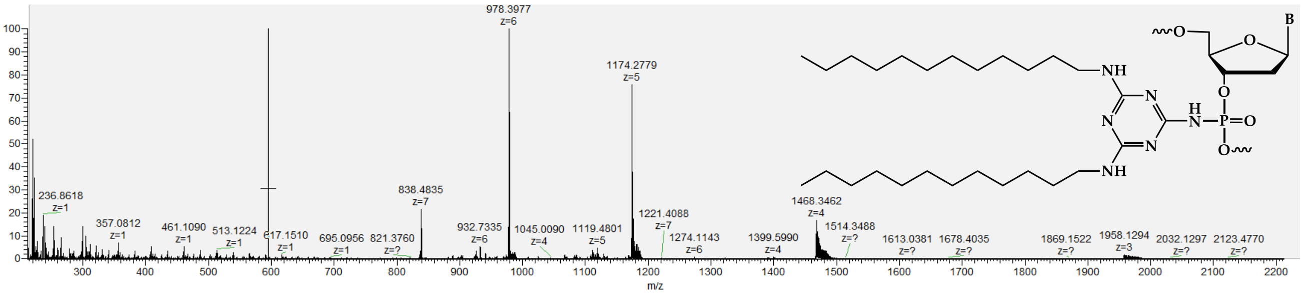

TO2 5′- [FAM]-TTT-TTT-T*T -3′,

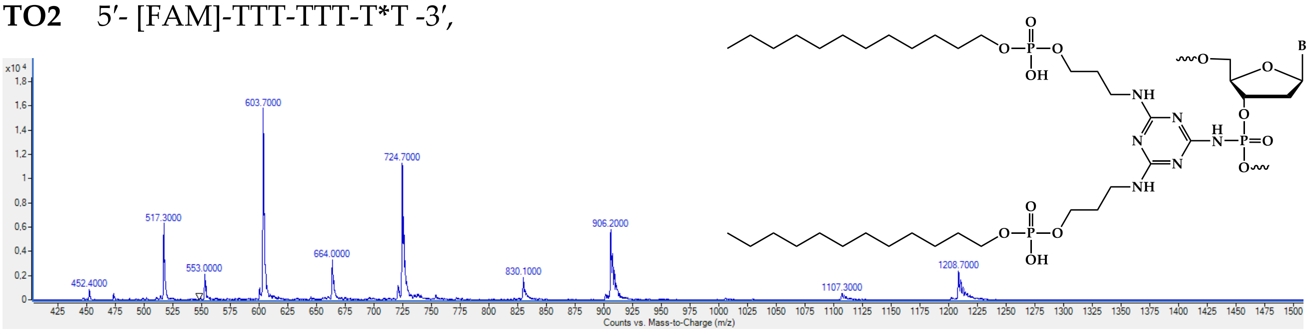

Figure S2. Undeconvoluted results of ESI mass spectrometry in negative ion registration mode of corresponding modifed oligonucleotides and structure of introduced lipophilic residues.

SUPPLEMENTARY MATERIAL S3

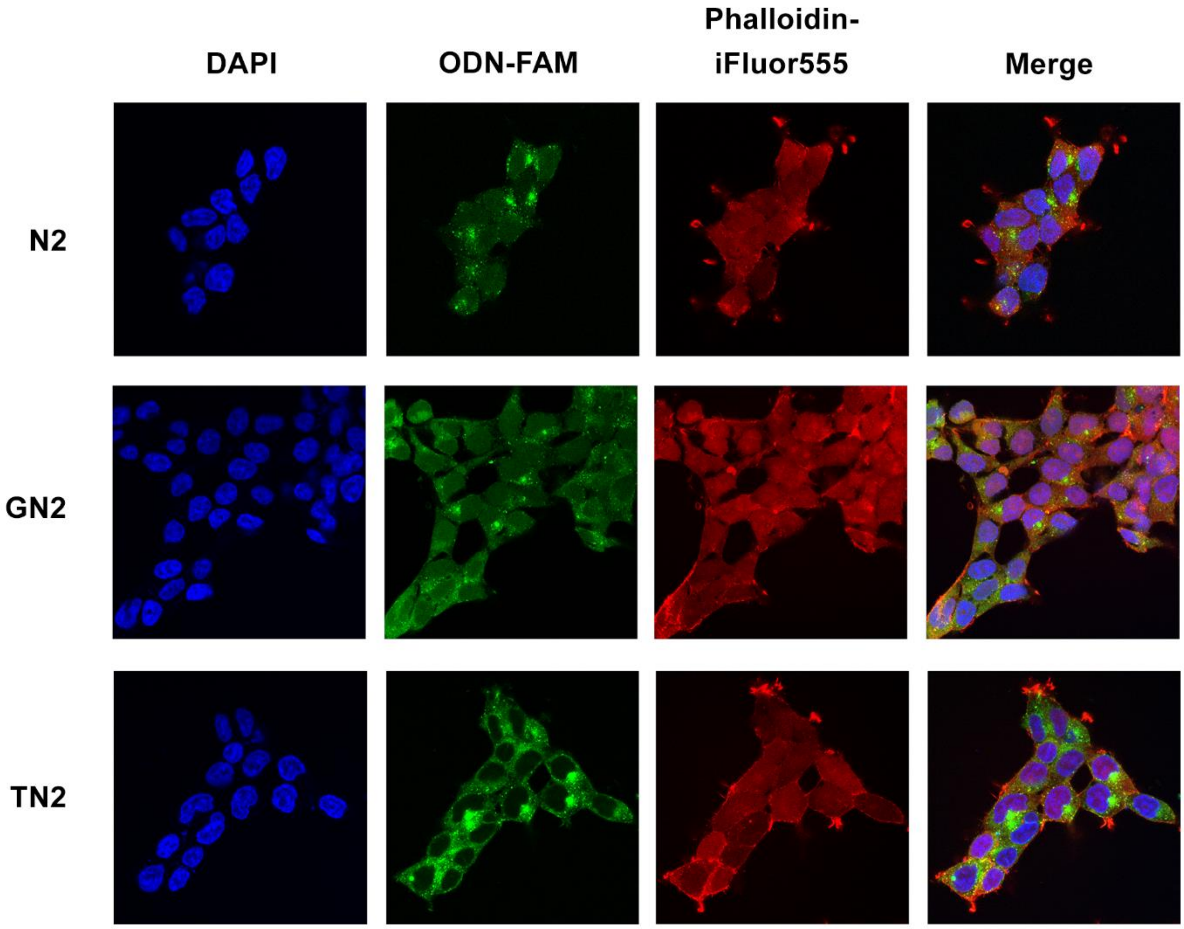

Figure S3. Intracellular accumulation of N2, GN2 and TN2 in HEK293T cells. Samples were analyzed 4 h after addition of FAM-labeled ODNs (5µM) to cells in serum-free medium. Analysis was performed with a Plan-Apochromat 63×/1.40 Oil DIC M27 objective in a LSM710 laser scanning confocal microscope (Carl Zeiss, Germany). Three-channel images were obtained by staining of nuclei with DAPI (blue channel) and cytoskeleton with Phalloidin-iFluor555 (red channel); FAM-labeled ODNs (green channel).

SUPPLEMENTARY MATERIAL S4

Table S1. Melting temperatures for duplexes of studied oligonucleotides with complementary unmodified oligonucleotide M, 5'-AAT ACT TCA TAG TCA G-3'. Thermal denaturation experiments were carried out using a UV detector on a UV-1800 UV spectrophotometer (Shimadzu, Japan) equipped with a Peltier block. Equimolar amounts (4 μM, 50 μL of each strand) of complementary oligonucleotides were used. Melting curves were recorded at 260 nm within a temperature range from 25°C to 85°C with a heating/cooling rate of 0.2°C/min, in a x1 PBS buffer.

Code	T_m, °C	Δ T_m, °C
O2 / M	53,1	+ 2.8
GN2 / M	51,0	+ 0.7
TN2 / M	49,8	- 0.5
C / M	50,3	–
